# Diffusion-Weighted Imaging Image Combined with Transcranial Doppler Ultrasound in the Diagnosis of Patients with Cerebral Infarction and Vertigo

**DOI:** 10.1155/2022/5313238

**Published:** 2022-06-30

**Authors:** Ying Lv, Yijie Zhang, Jun Wu

**Affiliations:** ^1^Department of Neurology and Psychiatry, Beijing Shijitan Hospital, Capital Medical University, Beijing 100038, China; ^2^Department of Emergency, Beijing Shijitan Hospital, Capital Medical University, Beijing 100038, China; ^3^Department of Community Medical Center, Beijing Shijitan Hospital, Capital Medical University, Beijing 100038, China

## Abstract

This study aimed at exploring the application value of diffusion-weighted imaging (DWI) combined with transcranial Doppler (TCD) in the diagnosis of patients with cerebral infarction and vertigo (CI + V). In this article, using a retrospective case-control study, 100 CI + V patients (CI + V group) were examined by DWI combined with TCD. Seventy cases of noncerebral infarction with vertigo (control group) who were hospitalized at the same time were collected for clinical data analysis and comprehensive evaluation of each index. The results showed that in patients with CI + V, the abnormal rate of blood vessels was proportional to the size of the lesion, and the abnormal rate of blood vessels in the large-area infarction group (97%) was much higher than that of the small-area infarct group (62%) and the lacunar infarction group (51%). The overall abnormal rate of blood vessels in the CI + V group (71%) was greatly higher than that in the control group (15%), showing a statistically and extremely great difference (*P* < 0.01). In short, DWI can effectively extract lesion-related data, and combined with TCD examination, the clinical diagnosis of CI + V can be more accurately performed, which had a positive impact on the clinical work of CI + V. This work provided some reference for the clinical effective diagnosis method of CI + V.

## 1. Introduction

Cerebral infarction is also known as ischemic stroke, and it is a common cerebrovascular disease (CVD). It is caused by the obstruction of blood supply to the brain tissue area, which leads to cerebral tissue ischemia and hypoxia, resulting in clinical brain tissue necrosis and corresponding neurological deficits [1, 2]. Most of the patients are middle-aged and elderly people, and the clinical cause is generally intracranial or cervical aortic atherosclerosis caused by a variety of factors [3, 4]. The early clinical symptoms mainly include dizziness, blackness, and numbness in different parts of the body, and there are life-threatening risks in the middle and late stages [5, 6]. Cerebral infarction is often accompanied by vertigo, which is a pseudo-vertigo caused by systemic diseases. The patient feels his body is wandering without obvious foreign objects or self-rotating sensation. For people with high incidence, the initial diagnosis and treatment of cerebral infarction are very necessary. Clinically, brain structure imaging and cerebrovascular imaging are usually used for diagnosis, which generally include cranial computed tomography (CT), and cranial magnetic resonance imaging (MRI), diffusion-weighted imaging (DWI), and transcranial Doppler ultrasound (TCD) [7].

Among them, cranial CT is the most convenient and commonly used. It can detect some subtle early ischemic changes within 6 hours of onset, but it is hard to distinguish after 2–3 weeks and lacks sensitivity to the posterior brainstem and cerebellum lesions [8]. Cranial MRI can clearly image these two parts, but it is difficult to detect lesions within a few hours of onset. The diagnostic detection rate of early acute CI + V by conventional CT and MRI is low with poor sensitivity, which can meet the requirements of clinical medical diagnosis and treatment. TCD uses blood flow velocity to assess the blood flow status, thereby inferring changes in local cerebral blood flow. It is a commonly used noninvasive inspection method to detect intracranial and extracranial vascular stenosis or occlusion [9, 10]. However, some researchers believe that the results of TCD are subjectively affected by the operator and are not as accurate as magnetic resonance angiography (MRA), digital subtraction angiography (DSA), and other invasive examination methods. DWI is an MRI function imaging technology. With the application of high field-strength MRI machine, it is the only MRI technology that can reflect the diffusion of water molecules and possesses high sensitivity. DWI can not only detect the early ischemic location, but also clearly show some small infarcts, which are mainly used for the diagnosis of ultra-early cerebral ischemia [11].

Therefore, in the above context, a retrospective investigation was conducted on CI + V patients who were examined jointly by DWI and TCD and 70 non-CI + V patients were hospitalized at the same time, to explore whether DWI combined with TCD has good clinical diagnostic value in CI + V, and to provide theoretical basis for the further promotion and application of this combined examination program.

## 2. Materials and Methods

### 2.1. Research Objects

A total of 100 patients hospitalized for CI + V from May 2018 to December 2020 in hospital were selected as subjects, including 60 males and 40 females, with an average age of 65 ± 10.5 years. Seventy cases of noncerebral infarction patients with vertigo were enrolled at the same time, including 44 males and 26 females, with an age range of 63.5 ± 9.7 years. All patients underwent head CT or MRI examination and were excluded from cerebrovascular diseases. There was no statistical significance in general clinical data with patients in the CI + V group. All subjects were divided into the CI + V group and the control group. The CI + V group was divided into three groups according to the lesion area presented by imaging examination. There were 31 patients with large-area cerebral infarction (>15 cm^2^), 46 patients with small-area cerebral infarction (<5 cm^2^), and 23 patients with lacunar cerebral infarction. The clinical data were analyzed by a retrospective case-control study. All the subjects agreed to sign informed consents, and this study had been approved by the ethics committee of hospital.

Inclusion criteria are as follows: the patients who were diagnosed according to the diagnostic criteria given in the 2010 Chinese Guidelines for the Diagnosis and Treatment of Ischemic Stroke; patients who received DWI in combination with TCD within 48 hours after admission; patients with no previous history of cerebral infarction; and patients without serious organic disease.

Exclusion criteria are as follows: patients with otogenic vertigo, ocular vertigo, or dizziness caused by psychological effects, emotional anxiety, and depression; patients with chronic subdural hematoma and other diseases; pregnant and lactating women; and unable to perform correlation analysis due to unknown medical records.

### 2.2. TCD Inspection Plan

All subjects were examined by a transcranial Doppler analyzer with a pulse probe frequency of 2 MHz. Through the examination, the subject's information was acquired, including anterior cerebral artery (ACA), middle cerebral artery (MCA), posterior cerebral artery (PCA), bilateral vertebral artery (VA), and basilar artery (BA). In addition, the average blood flow velocity (Vm), vascular pulsatility index (PI), peak systolic blood flow velocity (Vs), peak diastolic blood flow velocity (Vd), etc., were also determined

### 2.3. DWI Inspection Plan

1.5 T magnetic resonance scanner was used for TCD underwent cranial MRI, which was used to conventionally scan the transverse *T*2-weighted imaging (*T*2WI), *T*1-weighted imaging (*T*1WI), coronal *T*2WI, sagittal *T*1WI, DWI, and *T*2 ^*∗*^WI. The parameters were set as follows: DWI—repetition time was 5,000 ms, echo time was 90 ms, scanning layer thickness was 6 mm, layer spacing was 1 mm, matrix was 200 mm × 220 mm; and *T*2 ^*∗*^WI—repetition time was 450 ms, echo time was 2.53 ms, scanning layer thickness was 6 mm, layer spacing was 1 mm, and matrix was 220 mm × 200 mm.

### 2.4. Image Processing Scheme

Texture feature parameters in DWI images were extracted by full quantitative postprocessing software, which were read and analyzed by two experienced professional physicians. By standardizing and calibrating DWI images, the signal characteristics of each sequence position on the lesion in the CI + V group were observed. The image was segmented, and the relevant texture feature parameters were obtained. The apparent diffusion coefficient (ADC) values of all subjects in the relevant area were measured. All data were calculated automatically by computer software.

### 2.5. Statistical Analysis

SPSS 24.0 software was used for statistical analysis, the measurement data were expressed as mean ± standard deviation (‾*x* ± *s*), and the *χ*^2^ test was adopted for comparison between groups. The Kolmogorov–Smirnov test was applied to analyze whether the experimental data were consistent with the Gaussian distribution. If it was consistent, the *t*-test was used. *P* < 0.05 meant that the difference was statistically significant. Origin 8.0 was adopted for drawing.

## 3. Results

### 3.1. DWI Results of Typical Cases

**Figure 1:**
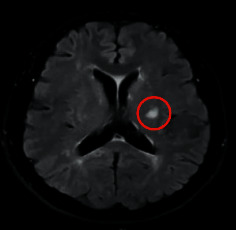
DWI imaging results of a typical case. The red circle marked the infarcted area.

A 55-year-old female patient presented with recurrent headache, dizziness, and limb weakness for more than 2 years, poor mobility, and worsening blurred vision for 3 weeks. On admission, DWI examination showed that the left lesion showed an obvious high signal, and the clinical diagnosis was acute cerebral infarction in the left basal ganglia with vertigo (Figure 1).

### 3.2. Comparison of Blood Vessel Conditions among the Subgroups of Patients in the CI + V Group

#### 3.2.1. Comparison of Blood Vessel Conditions of Patients between Large-Area Infarction Group and Small-Area Infarction Group

**Figure 2:**
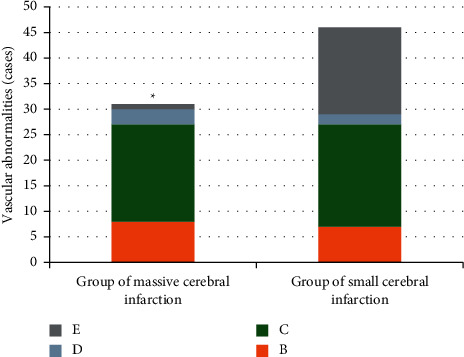
Comparison of blood vessel conditions of patients between large-area infarction group and small-area infarction group. *B*, *C*, *D*, and *E* in the figure referred to increased blood flow velocity, decreased blood flow velocity, without blood flow signal, and normal blood vessels, respectively.  ^*∗*^The difference in the abnormal rate of blood vessels of patients in the two groups was statistically obvious, *P* < 0.05.

As shown in Figure 2, there were 31 patients in the large-area infarction group and 46 patients in the small-area infarction group. Among them, there were 8 patients in the large-area infarction group and 7 patients in the small-area infarction group with increased blood flow velocity; and 19 cases were in the large-area infarction group and 20 cases were in the small-area infarction group with decreased blood flow velocity; there were 3 cases in the large-area infarction group and 2 cases in the small-area infarction group without blood flow signal.; and there were 1 case in the large-area infarction group and 17 cases in the small-area infarction group with normal blood vessels. The abnormal rate of blood vessels in the large-area and the small-area infarction groups was 97% and 62%, respectively. It suggested that the DWI combined with TCD examination showed that the abnormal rate of blood vessels of cerebral infarction was directly proportional to the size of the lesion, and the abnormal rate of blood vessels of the large-area infarction group was observably higher than that of the small-area infarct group, showing a statistically significant difference (*P* < 0.05).

#### 3.2.2. Comparison of Blood Vessel Conditions of Patients between Large-Area Infarction Group and Lacunar Infarction Group

**Figure 3:**
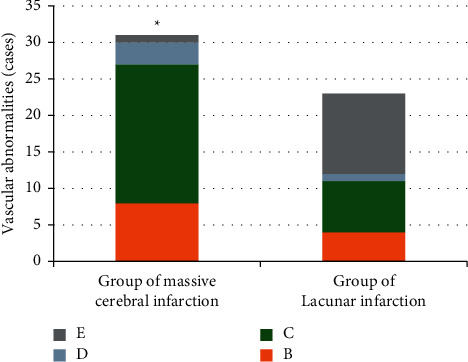
Comparison of blood vessel conditions of patients between large-area infarction group and lacunar infarction group. *B*, *C*, *D*, and *E* referred to increased blood flow velocity, decreased blood flow velocity, without blood flow signal, and normal blood vessels, respectively.  ^*∗*^The difference in the abnormal rate of blood vessels of patients in the two groups was statistically obvious, *P* < 0.05.

As shown in Figure 3, there were 23 patients in the lacunar infarction group, including 4 patients with increased blood flow velocity, 7 patients with decreased blood flow velocity, 1 patient without blood flow signal, and 11 cases with normal blood vessels. The abnormal rate of blood vessels in the lacunar infarction group was 51%. Thus, the abnormal rate of blood vessels in the large-area infarction group was visibly higher than that in the lacunar infarction group after DWI combined with TCD examination, and the difference was statistically obvious (*P* < 0.05).

#### 3.2.3. Comparison of Blood Vessel Conditions of Patients between Small-Area Infarction Group and Lacunar Infarction Group

**Figure 4:**
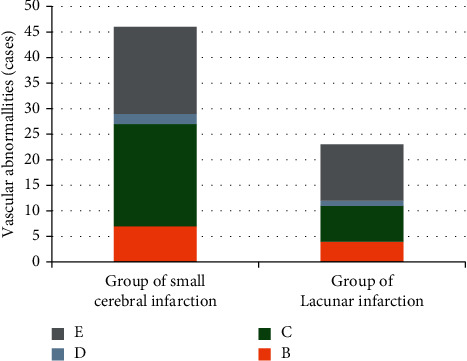
Comparison of blood vessel conditions of patients between the small-area infarction group and lacunar infarction group. *B*, *C*, *D*, and *E* referred to increased blood flow velocity, decreased blood flow velocity, without blood flow signal, and normal blood vessels, respectively.

As illustrated in Figure 4, the abnormal rate of blood vessels in the small-area infarction group was 62% and that in the lacunar infarction group was 51%. It indicated that the DWI combined with TCD suggested that the difference in the abnormal rate of blood vessels between the two groups was not statistically significant (*P* < 0.05).

### 3.3. Comparison on Blood Vessel Condition of Patients in the CI + V Group and the Control Group

**Figure 5:**
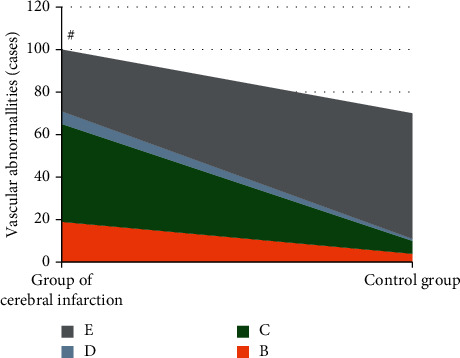
Comparison on the blood vessel condition of patients in the CI + V group and the control group. *B*, *C*, *D*, and *E* referred to increased blood flow velocity, decreased blood flow velocity, without blood flow signal, and normal blood vessels, respectively. #The difference in the abnormal rate of blood vessels of patients in the two groups was statistically obvious, *P* < 0.01.

As shown in Figure 5, there were a total of 100 patients with CI + V, including 19 cases with increased blood flow velocity, 46 cases with decreased blood flow velocity, 6 cases with no blood flow signal, and 29 cases with normal blood vessels. Thus, the abnormal rate of blood vessels was 71%. A total of 70 patients in the control group included 4 cases with increased blood flow velocity, 6 cases with decreased blood flow velocity, 1 case with no blood flow signal, and 59 cases with normal blood vessels, so the abnormal rate of blood vessels was 15%. The DWI combined with TCD examination showed that the abnormal rate of blood vessels in the CI + V group was much higher than that in the control group, and the difference was extremely and statistically obvious (*P* < 0.01).

### 3.4. Comparison of DWI Combined with TCD, CT, and MRI in Locating Lesions

**Figure 6:**
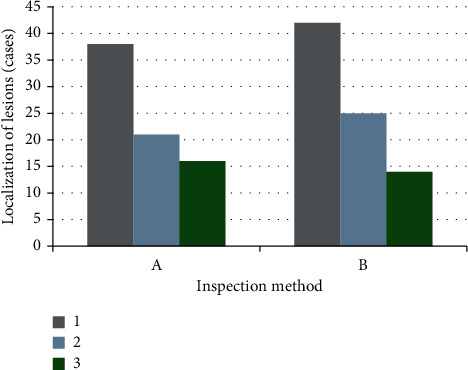
Comparison of DWI combined with TCD, CT, and MRI in locating lesions. A referred to the results of DWI combined with TCD; B showed the results of CT and MRI. 1, 2, and 3 referred to the number of patients with infarction of the internal carotid artery system, infarction of the vertebral base arterial system, and the ischemic infarction of the marginal zone between adjacent blood vessels, respectively.

**Figure 7:**
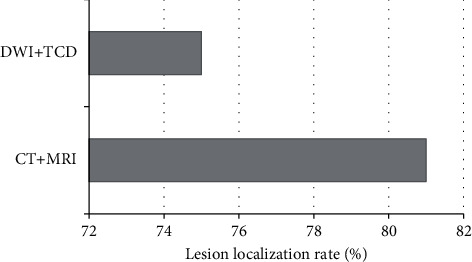
Comparison of lesion localization rates between the two methods.

As shown in Figures 6 and 7, among the 100 patients with cerebral infarction and vertigo, CT and MRI can locate the lesions in 81 cases. Among them, 42 cases had internal carotid artery system infarction, 25 cases had vertebrobasilar system infarction, and 14 cases had ischemic infarction in the border zone between adjacent blood supply areas. DWI combined with TCD could locate the lesions in 75 cases, including 38 cases of internal carotid artery system infarction, 21 cases of vertebrobasilar system infarction, and 16 cases of border zone ischemia between adjacent blood supply areas. It meant that there was no significant difference between DWI combined with TCD and CT and MRI in locating lesions (75% and 81%, respectively), and there was no statistical significance (*P* > 0.05).

### 3.5. DWI Combined with TCD to Detect the Blood Flow Velocity in the CI + V Group

**Figure 8:**
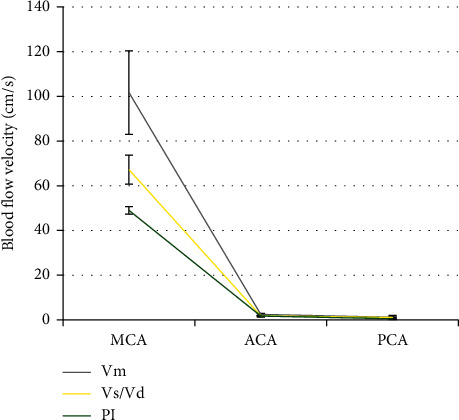
The specific conditions of patients with increased blood flow velocity.

**Figure 9:**
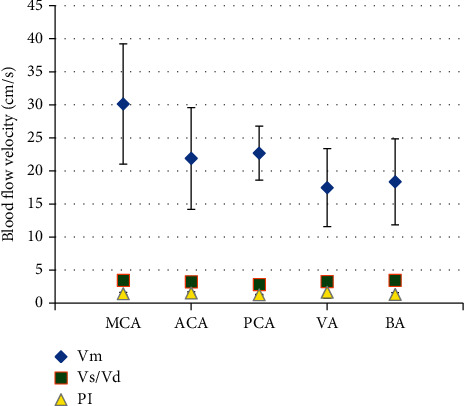
The specific conditions of patients with decreased blood flow velocity.

The blood flow velocity of patients in the CI + V group detected by DWI combined with TCD is illustrated in Figures 8 and 9. There were 19 cases with increased blood flow velocity and 46 cases decreased blood flow velocity.

For patients with increased blood flow velocity: in MCA, Vm was 101.7 ± 18.7 cm/s, Vs/Vd was 2.46 ± 0.5, and PI was 1.17 ± 0.8; in ACA, Vm was 67.2 ± 6.5 cm/s, Vs/Vd was 1.83 ± 0.2, and PI was 1.09 ± 0.24; and in PCA, Vm was 49 ± 1.7 cm/s, Vs/Vd was 1.7 ± 0.44, and PI was 0.49 ± 0.33.

For patients with decreased flood flow velocity: in MCA, Vm was 30.14 ± 9.1 cm/s, Vs/Vd was 3.46 ± 0.2, and PI was 1.43 ± 0.19; in ACA, Vm was 21.9 ± 7.7 cm/s, Vs/Vd was 3.24 ± 0.28, and PI was 1.53 ± 0.16; in PCA, Vm was 22.7 ± 4.08 cm/s, Vs/Vd was 2.82 ± 0.14, and PI was 1.27 ± 0.04; in VA, Vm was 17.46 ± 5.9 cm/s, Vs/Vd was 3.28 ± 0.61, and PI was 1.65 ± 0.9; and in BA, Vm was 18.36 ± 6.51 cm/s, Vs/Vd was 3.46 ± 0.77, and PI was 1.32 ± 0.28.

### 3.6. Composition of ADC Value of Patients in the CI + V Group and the Control Group

**Figure 10:**
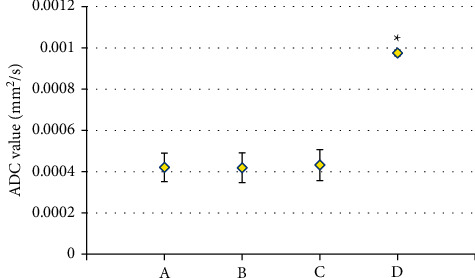
Composition of the ADC value of patients in the CI + V group and the control group. A, B, C, and D referred to the ADC values of patients in the large-area cerebral infarction group, small-area cerebral infarction group, the lacunar infarction group, and the control group, respectively.  ^*∗*^The ADC value between the CI + V group and the control group showed a statistically great difference, *P* < 0.05.

As shown in Figure 10, the patients in the CI + V group showed low ADC values; and ADC values of patients in the large-area cerebral infarction group, small-area cerebral infarction group, and the lacunar infarction group were 0.000421 ± 0.000069 mm^2^/s, 0.000419 ± 0.000072 mm^2^/s, and 0.000432 ± 0.000075 mm^2^/s, respectively. The ADC of patients in the control group was 0.000975 ± 0.000014 mm^2^/s. It suggested that the ADC values of the large-area cerebral infarction group, the small-area cerebral infarction group, and the lacunar infarction group were not statistically different (*P* > 0.05), while the difference in ADC values between the CI + V group and the control group was statistically significant (*P* < 0.05).

### 3.7. The Specific Situation of Patients with Clinical Vertigo

**Figure 11:**
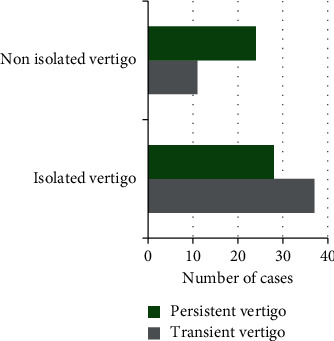
The specific situation of patients with clinical vertigo.

As revealed in Figure 11, there were 48 cases of isolated vertigo (including 37 cases of transient attacks and 11 cases of persistent attacks) and 52 cases of nonisolated vertigo (including 28 cases of transient attacks and 24 cases of persistent attacks).

**Figure 12:**
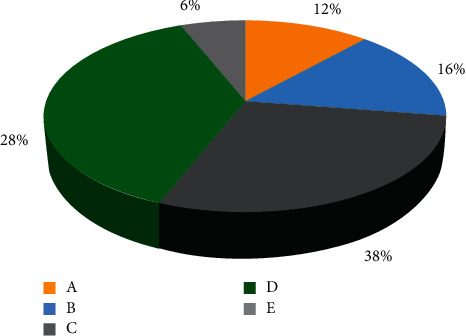
The specific symptoms of patients with clinical vertigo.

**Figure 13:**
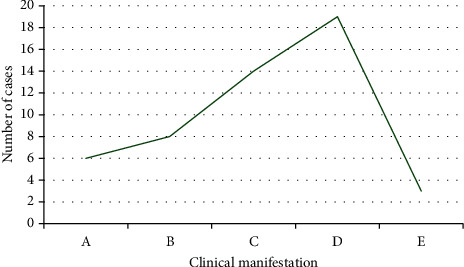
The specific symptoms of patients with clinical vertigo. A–E in the figure represented to the ambiguity, eyelid movement, body numbness, mild hemiplegia, and other symptoms, respectively.

During the experiment, 6 cases suffered from ambiguity, 8 cases suffered from eyelid movement, 14 cases suffered from body numbness, 19 cases suffered from mild hemiplegia, and 3 cases suffered from other symptoms (Figures 12 and 13).

## 4. Discussion

Cerebral infarction, also known as ischemic stroke, is a kind of cerebral blood circulation disorder. It is the most common type of cerebral vascular disease, which is caused by ischemia and hypoxia [12, 13]. It is more common in middle-aged and elderly patients. The general clinical diagnosis is based on brain structure imaging and cerebrovascular imaging. Among them, TCD is a noninvasive examination method used to evaluate the hemodynamics of the skull base arteries, which can more sensitively reflect the functional status of the cerebrovascular [14, 15], but it has some shortcomings, which is embodied that it fails to guarantee the incident angle of ultrasound, reducing the accuracy of repeated measurement of blood flow velocity. DWI can detect the size and location of the lesion in the ultra-early stage of onset, and it can also detect some small infarcts in the brainstem and cerebellum [16–18].

A retrospective study was conducted on 100 CI + V patients who underwent DWI combined with TCD examination to obtain effective data information from THEIR DWI images. The results showed that in patients with CI + V, the abnormal rate of blood vessels was directly proportional to the size of the lesion. The abnormal rate of blood vessels in the large-area infarction group was much higher than that in the small-area infarction group and the lacunar infarction group, showing statistically obvious differences (*P* < 0.05). In addition, the overall abnormal rate of blood vessels in the CI + V group was obviously higher than that in the control group, and the difference was extremely and statistically significant (*P* < 0.01). For patients with vertigo symptoms during the experiment, their clinical manifestations were also consistent with the diagnosis of vertigo. Takahashi [19] ever pointed out in the article that the success of the treatment of CI + V depended on whether the function of ischemic zone can be recovered as early as possible. However, traditional CT and MRI were both unable to accurately determine the location and scope of lesions at early stage. Consequently, clinical diagnosis becomes difficult. DWI could diagnose CI + V accurately, thus guiding clinical treatment, which was consistent with the conclusion of the research. Snider et al. [20] demonstrated the values of TCD in diagnosing and treating CI + V. TCD showed high accuracy of diagnosing CI + V with quick and convenient operation as well as no trauma. TCD helped people understand the functional state of intracranial vascular preliminary. The examination results showed significant guidance meaning for the early diagnosis and assessment of CI + V.

## 5. Conclusion

A retrospective study was conducted on 100 CI + V patients who underwent DWI combined with TCD examination. It was found that the information of the cerebral infarction lesions extracted suggested that in patients with CI + V, the abnormal rate of blood vessels in the large-area infarction group was obviously higher than that in the small-area infarction group and the lacunar infarction group, showing statistical differences (*P* < 0.05); and compared with the control group, the overall abnormal rate of blood vessels in the CI + V group was highly statistically significant (*P* < 0.01). The deficiency is that there are only a few samples included in the final study due to the different quality level and degree of perfection of each case. Moreover, as this study is a retrospective study, there are many uncontrollable factors, so the results have certain limitations. In future studies, the sample size will be expanded to further explore this topic. In conclusion, DWI combined with TCD has a good application value in the diagnosis of CI + V, providing a theoretical basis for the clinical work of CI + V.

## Data Availability

The data used to support the findings of this study are available from the corresponding author upon request.
